# Rodent brain extraction and dissection: A comprehensive approach

**DOI:** 10.1016/j.mex.2023.102516

**Published:** 2023-12-07

**Authors:** Refat Aboghazleh, Silvia D. Boyajian, Afnan Atiyat, Manal Udwan, Mimas Al-Helalat, Renad Al-Rashaideh

**Affiliations:** a Department of Basic Medical Sciences, Faculty of Medicine, Al-Balqa Applied University, The College of Medicine Building, Al-Salt 19117, Jordan; b Department of Medical Neuroscience, Faculty of Medicine and Brain Repair Center, Dalhousie University, Halifax, Canada

**Keywords:** Brain hemispheres, Isolation brain regions, Rat brain, Brain extraction and dissection

## Abstract

The neuroscience is continuously expanding field, and conducting experiments serves as one of the most effective approaches to enhance and broad our understanding of this fascinating field. Most of the lab work in neuroscience involves the use of animal models such as rats and mice for experiments dedicated to monitoring cerebral changes.

The study:

• Introduces a practical method for brain extraction without perfusion with paraformaldehyde prioritizing brain integrity and avoiding damage.
• Offers a detailed, step-by-step dissection guide for different brain regions, including the hippocampus, cerebral cortex, corpus striatum, thalamus, cerebellum, and medial prefrontal cortex, from rodent brains, accompanied by high-resolution images that provide anatomical clarity.
• Presents enhanced reliability, precision, and detailed anatomical descriptions.
  *Conclusion*: This study has introduced a reliable technique for brain extraction that eliminates the need for paraformaldehyde perfusion. Furthermore, a comprehensive methodology has been presented for extracting different brain regions from rodent brains.

## Graphical abstract

**Figure:**
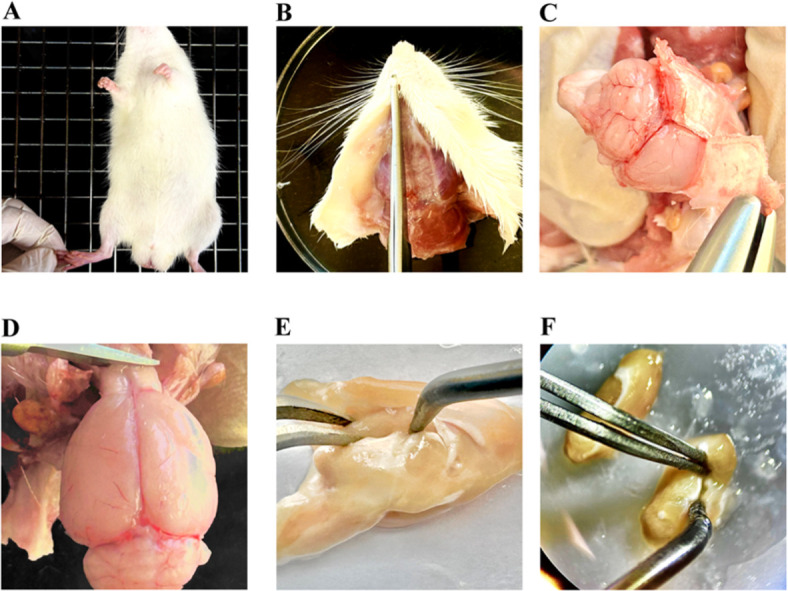

Specifications table

**Table:** 

Subject area:	Neuroscience
More specific subject area:	Methods in Neuroscience
Name of your method:	Brain extraction and dissection
Name and reference of original method:	Not applicable
Resource availability:	Not applicable

## Introduction

**Fig. 1:**
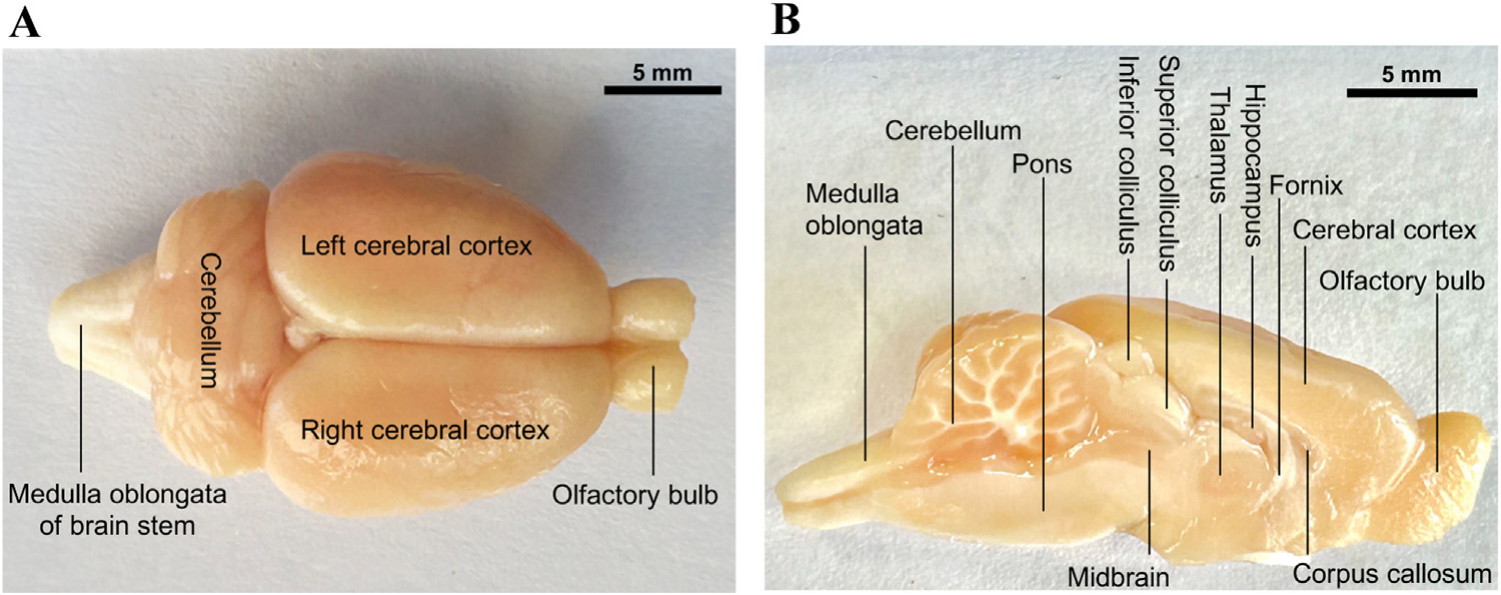
Gross anatomy of a rat brain. (A) Superior view of young adult rat brain. (B) Left sagittal view of the rat brain.

The field of neuroscience has experienced remarkable growth, with researchers increasingly turning to animal models, particularly rats and mice, as a valuable means to conduct experiments. Due to ethical and practical constraints, experimentation on humans is often unfeasible. Consequently, extracting the brains of rodents is a necessity to explore the effects of various treatments and evaluate the anatomical, histological, and physiological changes at both gross and microscopic levels [1,5]. Furthermore, examining brain tissue through immunohistochemistry, polymerase chain reaction (PCR) analysis, and diverse staining techniques such as silver stain, Golgi stain, kajal stain, and hematoxylin and eosin stain allow for comprehensive investigations. Although few brain extraction methods exist, selecting the anatomically optimal approach ensures the preservation of brain tissue integrity and facilitating subsequent analyses [4]. Notably, certain research objectives, such as preserving RNA for PCR, require immediate dissection of specific regions immediately after extraction without paraformaldehyde perfusion, including the hippocampus, cerebral cortex, corpus striatum, medial prefrontal cortex, thalamus, brain stem, cerebellum and other key areas. This paper aims to present the most effective and time-efficient method for brain extraction and dissection, enabling researchers to examine distinct brain regions (Fig. 1A and B) with precision and accuracy.

## Procedure

All procedures were performed following institutionally approved protocols in accordance with the Institutional Review Board at Al-Balqa Applied University, No. 26/3/1/1440.

### Instruments and equipment

- Small animal decapitator guillotine (Stoelting Co., No. 51330).
- Straight scissors (Fine Science Tools, No. 14012-15).
- Forceps, fine tip, curved (F. S. T, No. AB034).
- Halsted-mosquito forceps.
- Straight scissors with different sizes.
- Curved serrated forceps (F. S. T, No. 11051-10).
- Fine scissor (F. S. T, No. 14160-10).
- Micro scissor (F. S. T, No. 15000-03).
- Micro forceps (F. S. T, No. 11252-00 and 11274-20).
- Scalpel handle (size 3) and surgical blade (gauges 10 and 15).
- Boehler bone cutter (F. S. T, No. 16130-15).
- Stainless steel tray.
- Artificial cerebrospinal fluid (ACSF), in mmol is: KCl: 3, NaCl: 124, MgSO₄: 2, CaCl₂: 2, NaHCO₃: 26, glucose: 10, at 34-36 °C, pH 7.4, osmolarity 300 ± 10 mOsmol/l and carbonated with 95 % O₂ and 5 % CO₂ [3,5].
- Normal saline (0.9 % saline).
- Petri dish.
- Dissecting microscope (StereoBlue).
- Double edge razor blades.
- Transport plastic tubes (Citotest scientific, No. EN14254).
- Pointed curved tweezer.
- Pointed curved probe.
- Two flat ice packs.
- Sterile cotton swabs.
- Ruler.
- Alcohol (70 %).
- Syringe (1 ml and 3 ml).
- Isoflurane, Isoflurane vaporizer machine and 99.9 % oxygen tank.
- Ketamine and Xylazine.
- Eppendorf tubes (Fisher scientific, No. 0540294).

### Brain extraction procedure

1. Sedate the male rat in isoflurane induction chamber (2-4 %) for about 2–4 min. Once the animal is sedated, position the rat in supine position and deeply anesthetized the animal by injecting Ketamine and Xylazine (80 mg/kg and 10 mg/kg, respectively) intraperitoneally. Alternatively, pentobarbital (50 mg/kg, intraperitoneally) can be also given instead of Ketamine and Xylazine.
2. Return the rat back into its cage. Then, at intervals of approximately three minutes, check for sedation by assessing the absence of the toe-pinch reflex in the animal (Fig. 2A). If the rat does not exhibit signs of sedation, an additional half dose of Ketamine can be given intraperitoneally.

**Fig. 2:**
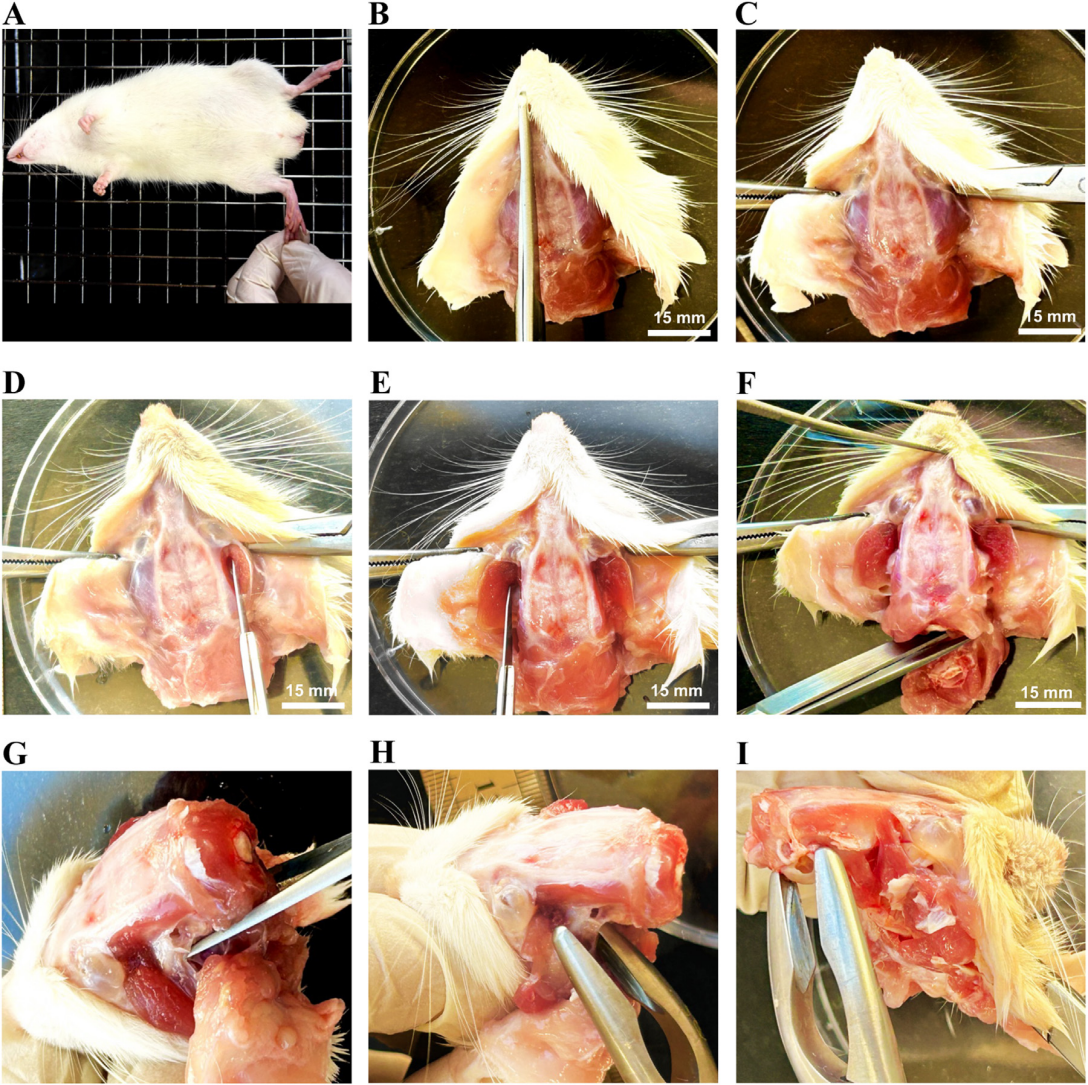
Rat brain extraction: scalp incision and muscle dissection procedure. (A) The animal was deeply anesthetized and checked for sedation by assessing the absence of the toe-pinch reflex. (B) Decapitated head were positioned, and a midsagittal incision made in the scalp. (C) Both sides of the scalp were retracted to expose the skull. The temporalis muscles were cut from both sides to expose the temporal bones of the skull (D and E). The cervical region was disconnected from the cranium, revealing the spinal cord emerging through the foramen magnum (F). The muscles of the pharynx and those associated with the basal part of skull were incised (G), and the ramus of the mandible was dislocated and removed (H). The bilateral tympanic bullae, situated on each side of the skull, were excised.
3. Once the animal is deeply anesthetized and there is a complete lack of reflexes, proceed to insert the rat's head and the initial portion of its neck into the decapitator. Subsequently, take off the head along with approximately 1 cm of the neck.
4. Using a straight scissor, cut the scalp from the midline from backward forward. Then, grasp each half of scalp by Halsted-mosquito forceps (Fig. 2B and C).
5. Expose each side of the skull by cutting each temporalis muscle on each side by the surgical blade and removed them (Fig. 2D and E).
6. Cut the proximal end of the neck and vertebrae from the occipital bone of the skull by the surgical blade or by using a straight scissor (Fig. 2F). Remove any remaining parts of muscles attached to the posterior and inferior parts of the skull using forceps, Boehler bone cutter, and serrated forceps (Fig. 2G).
7. Using the Boehler bone cutter, cut the zygomatic arches, coronoid process, and angular process of mandible on each side (Fig. 2H).
8. Create a small window in the inferolateral part of the skull by grasping and extracting the tympanic bulla on each side of skull (Fig. 2I) using serrated forceps or the Boehler bone cutter.
9. Cautiously, grasp the foramen magnum by Boehler bone cutter, with caution not to injure the spinal cord and proximally the brain stem. Cut the bone there and take off each part separately (Fig. 3A). The occipital bone will be removed as well. At this stage, the brain stem and the cerebellum are exposed (Fig. 3B and C).

**Fig. 3:**
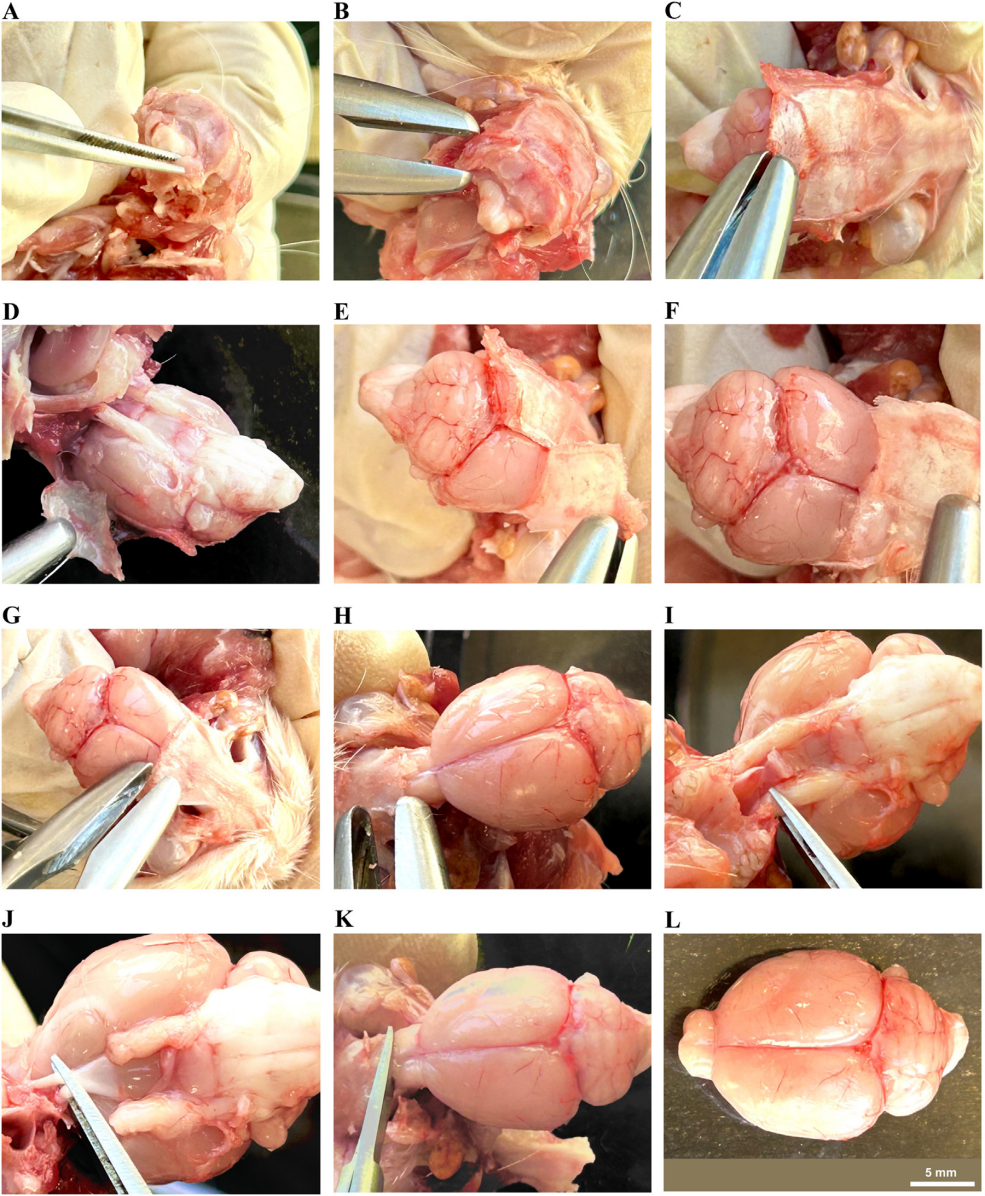
Rat brain extraction: cranial bones dissection. Dissection of the occipital bone forming the foramen magnum (A and B) and the part covering the cerebellum (C). The temporal bones on each sides were removed (D). The parietal bones covering the posterior part of the cortex were removed (E and F). The toughest and most anterior part of the skull (frontal bones) were carefully trimmed using a Boehlerbone cutter until the olfactory bulbs were exposed (G and H). At the inferior surface of the brain, the trigeminal nerves (I) and optic nerves (J) were cut. The olfactory bulbs were dissected (K). Finally, the brain was released and extracted (L).
10. Using the Boehler bone cutter cut and extract part of the basal part of the skull, making temporal and parietal bones ready to be removed.
11. Insert less than 1 mm of the tip of the Boehler bone cutter between the lateral side of the brain and the rest of temporal bone and take off as much as possible from that bone (Fig. 3D), with extra caution not to create any pressure on the brain itself. Repeat that to the other side of the brain.
12. Gently, pull up the parietal bones from the back up and take them off (Fig. 3E and F). At this stage, parietal bones will be removed with part of temporal bones as well. Most of the cerebral cortex now exposed.
13. The frontal bone is a tough bone. Similarly, insert less than 1 mm of the tip of the Boehler bone cutter between the frontal bone superiorly and the cortex of the brain beneath it and start to trim from that frontal bone without creating a pressure on the cortex of the brain (Fig. 3G). Continue until you expose the olfactory bulbs (Fig. 3H).
14. While the superior surface of brain is relieved, then move back to the inferior surface of the brain and cut the most prominent nerves there, including trigeminal and optic nerves (Fig. 3I and J, respectively). At this stage, the brain still connected from its tip by olfactory bulbs.
15. Using a laboratory spatula or micro scissor, separate the olfactory bulbs, and the brain is free to be extracted (Fig. 3K and L).

### Brain dissection procedure

1. Clean the ice packs, the dissecting microscope, and the equipment using 70 % ethanol. For solidifying and rigidity purposes, the rat brain, dissection equipment, and the stage of dissection should be kept cold by keeping them on ice packs during the procedure.
2. Put a Petri dish on the ice pack located on the dissection stage of the microscope.
3. Place the brain on its ventral side in the Petri dish.

**Fig. 4:**
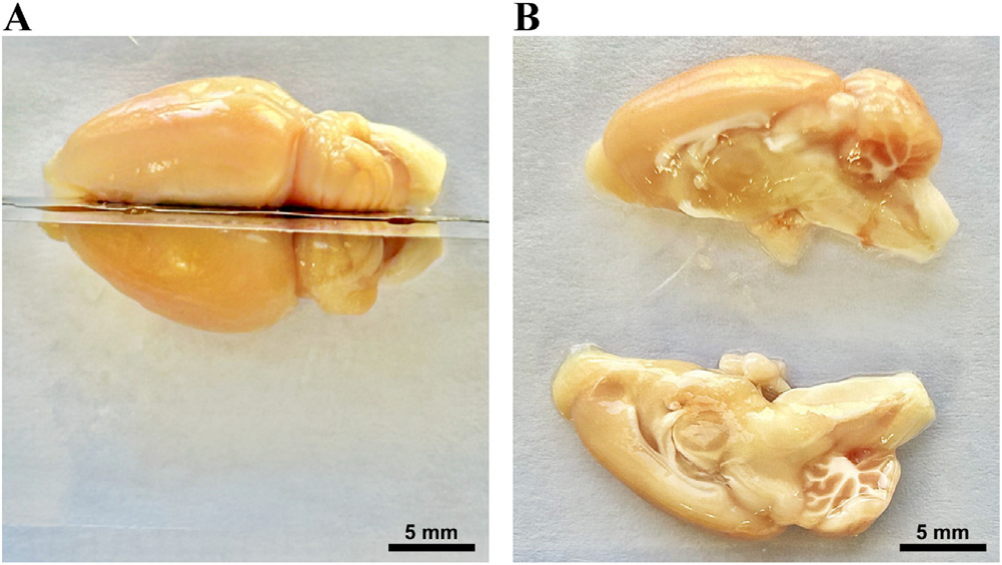
Rat brain dissection: division into two halves. The rat brain was meticulously dissected along the midsagittal plane using a razor blade (A), resulting in the division of the brain into two equal halves, the right and left hemispheres (B).
b. After cutting, use a normal saline or he ACSF to help the right and left hemispheres separating from each other (Fig. 4B).
c. Discard the excess normal saline solution or ACSF and use the swab to wipe any extra fluid. Assuming that the left side will be dedicated to the molecular study, all subsequent procedures will be relevant to the left-brain hemisphere.

*Dissection of brain hemispheres:*

a. Under the dissecting microscope place the razor blade accurately at the midline of the cerebrum (frontally) and the midline of the brainstem (posteriorly) to determine the precise location for cutting (at midsagittal plane) (Fig. 4A). Steady the brain with your left-hand fingers and move the razor carefully front and back with your right hand (if you are a right-handed person) until the razor reaches the bottom (the Petri dish).

**Fig. 5:**
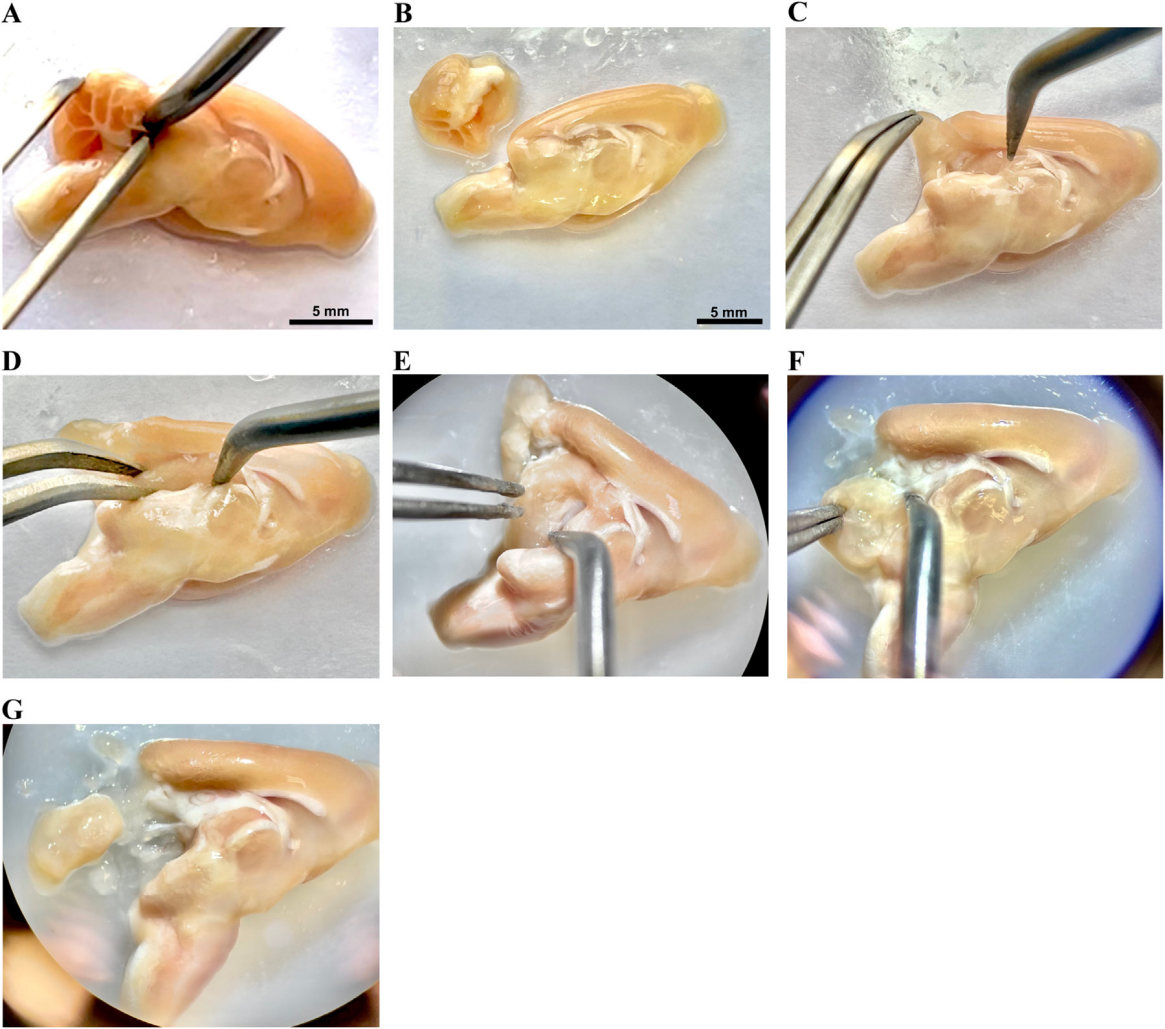
Rat brain dissection: Cerebellum and hippocampus. The cerebellum was carefully separated from the brain stem (A and B) and entirely extracted. (C) The cortex was gently reflected to exposed the superior part of the hippocampus. The hippocampus was cautiously separated from the surrounding structures, including the cortex, corpus callosum, and thalamus (D–F). The extracted hippocampus devoid of any cortical part connected to it (G).
c. Transfer the cerebellum to a sterile Eppendorf tube (using the tweezer) and label it.

*Cerebellum isolation:*

a. Flip up the left-brain half so the lateral side faces the Petri dish, and the medial side faces up.
b. Catch the cerebellum kindly using the pointed curved tweezer with the lefthand, and using the right hand, try to separate the cerebellum gently using the probe by isolating it from colliculi of midbrain, pons and medulla oblongata without rupturing the cerebellum or any of the brainstem parts (Fig. 5A and B).

*Hippocampus isolation:*

The hippocampus is located about 10 mm posteriorly from the tip of the brain [2]. The subsequent instructions outline the process of isolating the hippocampus:

a. Start from the back side midline (anterior to the extracted cerebellum) and carefully take the cortex up by your left hand using the pointed curved tweezer, without rupturing it. Simultaneously, the right hand has to hold the probe to separate the cortex without removing it (Fig. 5C and D). The initial, white-colored part encountered is most likely the corpus callosum, under it is the hippocampus.
b. Isolate the hippocampus carefully using the probe, trying not to rupture it (Fig. 5E–G). It is C-shaped structure located between the cortex externally and the thalamus internally.
c. Remove any part of the cortex connected to it.
d. Transfer it to a sterile Eppendorf tube (using the tweezer) and label it.

*Thalamus extraction:*

**Fig. 6:**
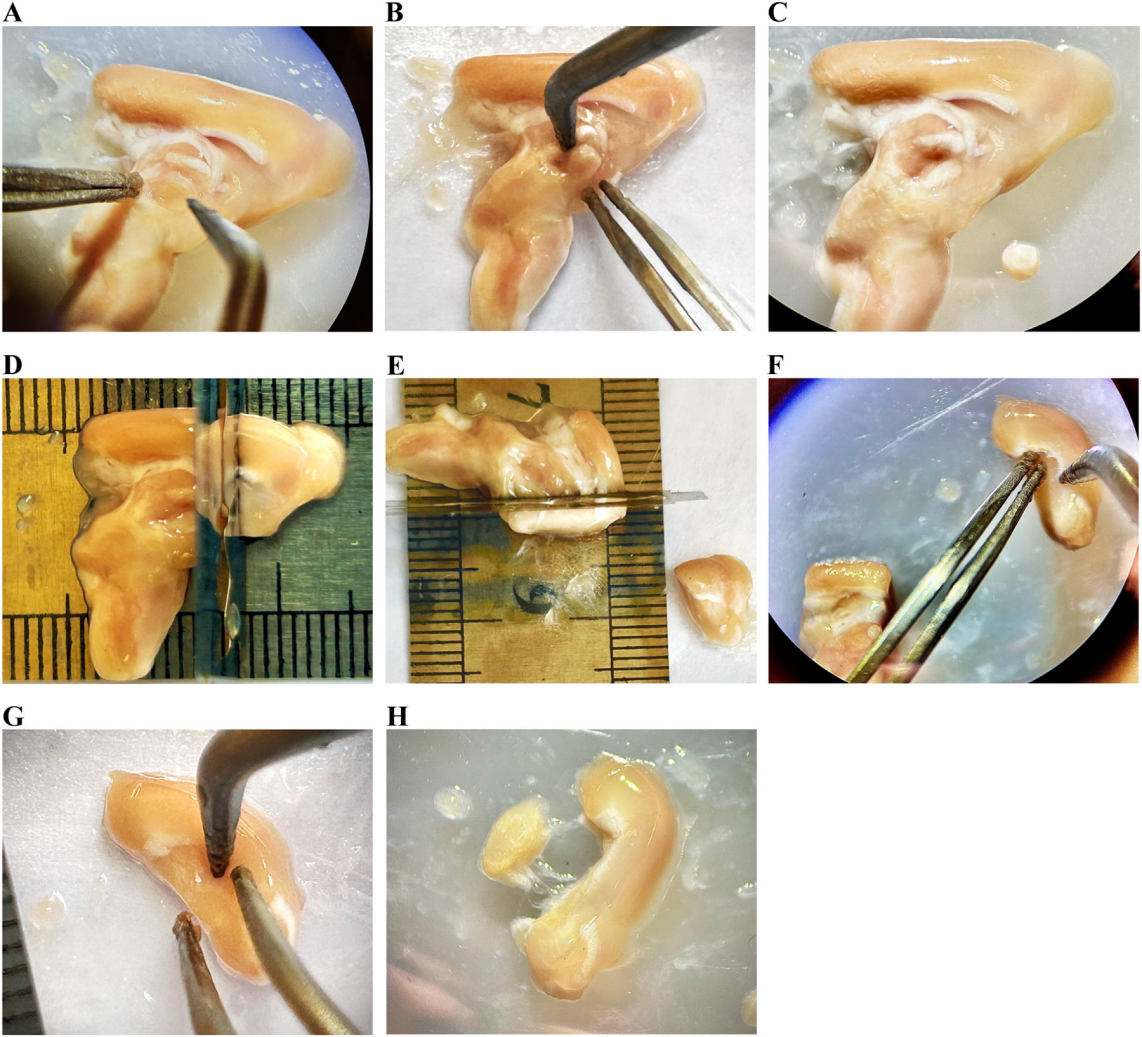
Rat brain dissection: Extraction of thalamus and corpus striatum. The thalamus was extracted from the medial side of the left hemisphere (A–C). A 3 mm section was cut from the anterior part of the rat brain, measured from the brain tip, excluding the olfactory bulbs (D), followed by cutting another 2 mm section (E) immediately posterior to the first cut. (F) The 2 mm brain section containing the corpus striatum. (G and H) The corpus striatum was separated from the corpus callosum and extracted.

Medial and inferior to the hippocampus, located about 8 mm from the tip of the brain [2], an oval-shaped structure which is bordered anteriorly by a bundle white matter fibers known as the fornix, this is the thalamus. As described previously, using the probe with your right hand while the left hand steady the brain using the tweezer, extract the thalamus and put it in a sterile Eppendorf tube and label it (Fig. 6A–C).

*Corpus striatum isolation:*

The corpus striatum is located about 3 mm from the tip of the brain [2] and spans about 2 mm, 3–5 mm from the tip of the brain. The following steps demonstrate the procedure for extracting the corpus striatum:

a. Place the ruler under the Petri dish.
b. Place the tip of the brain anteriorly at 0 mm (exclude olfactory bulbs).
c. Measure about 3 mm from the front and cut using the razor blade (Fig. 6D).
d. Measure about 2 mm from the last cut and proceed to make a second cut (Fig. 6E). The resulting 2 mm segment encompasses the corpus striatum, which is identifiable by its darker color and distinctive a drop-like structure (Fig. 6F).
e. Isolate the corpus striatum using the probe, excluding the white matter tract (corpus callosum) superior to it (Fig. 6G and H).
f. Take the corpus striatum and put it in a sterile Eppendorf tube and ensure it is properly labeled.

**Fig. 7:**
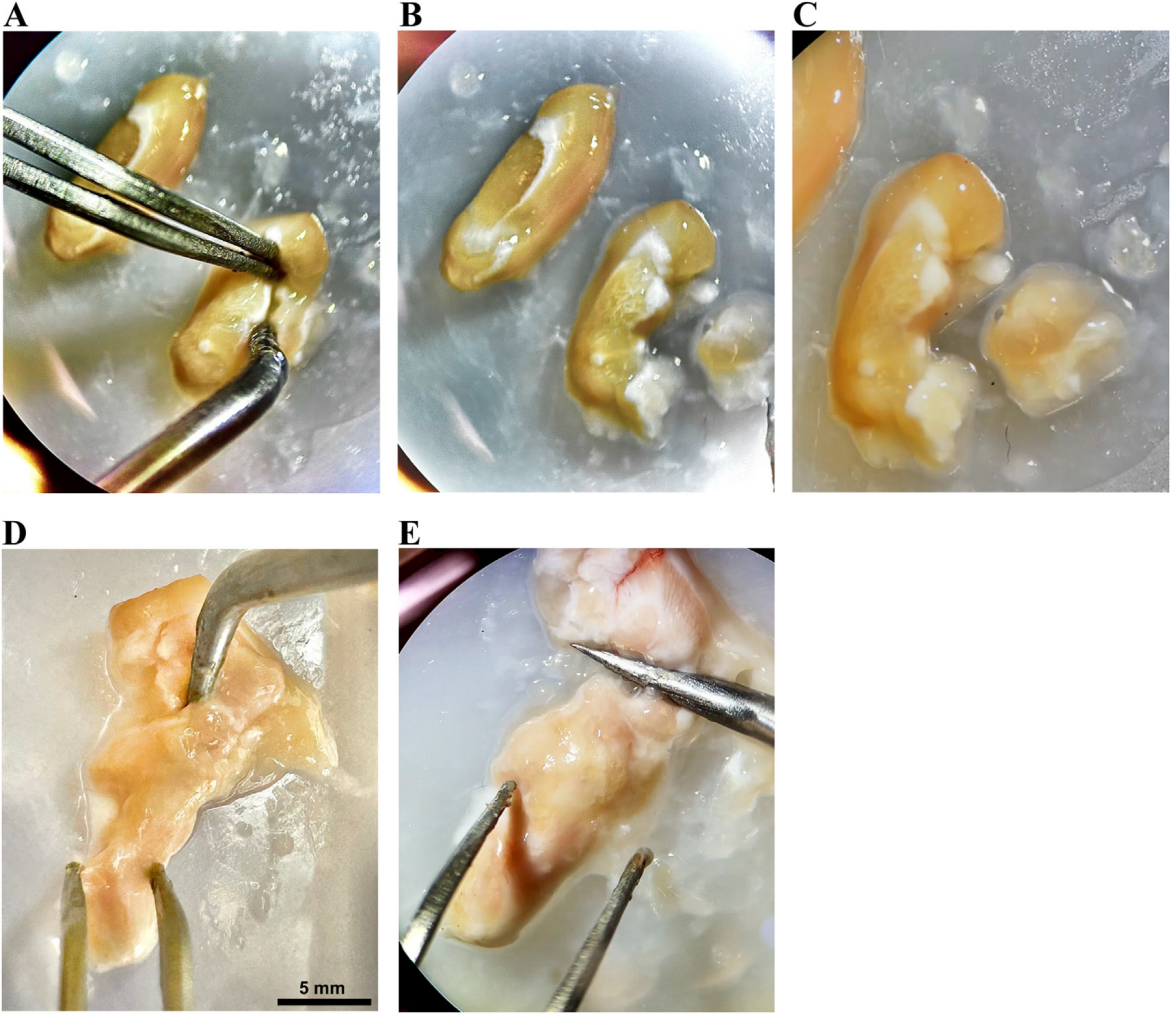
Medial prefrontal cortex extraction and isolation of brain stem in rat brain dissection. Extraction of the medial prefrontal cortex from the first 3 mm frontal segment (A, B, and C).The brain stem was separated by making an initial cut superiorly from the region anterior to superior colliculus of the midbrain downward obliquely to meet the pituitary gland inferiorly (D and E).
b. After isolating the medial prefrontal cortex, transfer it into a sterile Eppendorf tube and proceed to label it appropriately.

*Medial prefrontal cortex isolation:*

a. This area is extended from 0.4 to 1 mm from the midline laterally, and spans from 1 to 4 mm from the brain's tip [2]. It can be isolated from the first 3 mm frontal previous segment (Fig. 7A). It's likely to be the small area appearing at the midline. In that section of the brain, a major part of it is located between the midline medially and forceps minor corpus callosum and ventral orbital cortex laterally (Fig. 7B and C).

*Brainstem isolation:*

a. Put the probe in the front of the superior colliculus and at the back of the cortex using the right hand while the left hand has to steady the remaining brain (Fig. 7D).
b. The best way to exclude tissues from thalamus and hypothalamus is to start the dissection anterior to superior colliculus of the midbrain superiorly and cut obliquely to meet the pituitary gland inferiorly.
c. Complete this separation process by moving the probe down to separate the brainstem from the remaining brain (Fig. 7E).
d. Isolate it then put it in a sterile Eppendorf tube and label it.

*Cortex isolation:*

a. Take the uppermost external part of the previous 2 pieces (corpus striatum and medial prefrontal cortex extracted pieces), excluding the corpus callosum.
b. Take the previous pieces of cortex that surrounded the hippocampus as well.
c. Put it in a sterile Eppendorf tube and label it.

## Ethics statements

All procedures were performed following institutionally approved protocols in accordance with the Institutional Review Board at Al-Balqa Applied University, No. 26/3/1/1440.

## CRediT authorship contribution statement

**Refat Aboghazleh:** Conceptualization, Writing – original draft, Supervision, Writing – review & editing, Funding acquisition. **Silvia D. Boyajian:** Writing – original draft, Methodology, Writing – review & editing. **Afnan Atiyat:** Methodology, Writing – review & editing. **Manal Udwan:** Writing – original draft, Methodology, Writing – review & editing. **Mimas Al-Helalat:** Methodology, Writing – review & editing. **Renad Al-Rashaideh:** Methodology, Writing – review & editing.

## Declaration of Competing Interest

The authors declare that they have no known competing financial interests or personal relationships that could have appeared to influence the work reported in this paper.

## Data Availability

No data was used for the research described in the article.
